# Structure and Properties of Biodegradable PLLA/ZnO Composite Membrane Produced via Electrospinning

**DOI:** 10.3390/ma14010002

**Published:** 2020-12-22

**Authors:** Daria A. Goncharova, Evgeny N. Bolbasov, Anna L. Nemoykina, Ali A. Aljulaih, Tamara S. Tverdokhlebova, Sergei A. Kulinich, Valery A. Svetlichnyi

**Affiliations:** 1 Laboratory of Advanced Materials and Technology, Tomsk State University, Tomsk 634050, Russia; v_svetlichnyi@bk.ru; 2 Laboratory of Hybrid Plasma Systems, National Research Tomsk Polytechnic University, Tomsk 634050, Russia; ebolbasov@gmail.com (E.N.B.); aramat_tts@mail.ru (T.S.T.); 3 Laboratory of Biopolymers and Biotechnology, Tomsk State University, Tomsk 634050, Russia; nemoykina@rambler.ru; 4 Department of Mechanical Engineering, Tokai University, Hiratsuka, Kanagawa 259-1259, Japan; 5 Division of Physical Science and Engineering, King Abdullah University of Science and Technology (KAUST), Thuwal 23955-6900, Saudi Arabia; ali.julaih@kaust.edu.sa; 6 Research Institute of Science and Technology, Tokai University, Hiratsuka, Kanagawa 259-1259, Japan; 7 School of Natural Sciences, Far Eastern Federal University, Vladivostok 690091, Russia

**Keywords:** PLLA/ZnO composite membranes, ZnO nanoparticles, pulsed laser ablation, antibacterial properties

## Abstract

These days, composite materials based on polymers and inorganic nanoparticles (NPs) are widely used in optoelectronics and biomedicine. In this work, composite membranes of polylactic acid and ZnO NPs containing 5–40 wt.% of the latter NPs were produced by means of electrospinning. For the first time, polymer material loaded with up to 40 wt.% of ZnO NPs (produced via laser ablation in air and having non-modified surface) was used to prepare fiber-based composite membranes. The morphology, phase composition, mechanical, spectral and antibacterial properties of the membranes were tested by a set of analytical techniques including SEM, XRD, FTIR, UV-vis, and photoluminescence spectroscopy. Antibacterial activity of the materials was evaluated following standard procedures (ISO 20743:2013) and using *S. aureus* and *E. coli* bacteria. It is shown that incorporation of 5–10 wt.% of NPs led to improved mechanical properties of the composite membranes, while further increase of ZnO content up to 20 wt.% and above resulted in their noticeable deterioration. At the same time, the antibacterial properties of ZnO-rich membranes were more pronounced, which is explained by a larger number of surface-exposed ZnO NPs, in addition to those embedded into the bulk of fiber material.

## 1. Introduction

Biodegradable polymers and their composites are widely used in diverse areas of human activities, from prototyping to packaging and biomedicine. One of the most commonly used biodegradable polymers is polylactide or poly-L-lactic acid (PLLA) whose biomedical applications cover transplanting materials, those for regenerative medicine, wound dressing, targeting delivery, and so on [1]. For more efficient use of such polymers, their modification is often necessary to provide them with required functional properties [2]. For instance, addition of Ca and P compounds or antibacterial agents and microelements render such biomedical materials with properties that promote bone tissue regeneration or faster wound healing. Diverse functional additives or modifiers can be either deposited on the surface or incorporated into the bulk of PLLA, in the latter case the effect of prolonged functional effect being realized [3].

Recently, because of the wide spread of antibiotic-resistant bacteria, for antibacterial use, in addition to traditional antibiotics, inorganic and hybrid nanoparticles (NPs) are extensively studied and tested as such NPs are known not to allow bacteria to develop resistance. In this respect, along with traditionally and long-used Ag NPs, those of ZnO are probably the most popular antibacterial nanomaterial [4,5,6,7].

Thus, ZnO NPs can be considered promising for use in biomedical materials and medications. Furthermore, it is worth mentioning that Zn is one of important microelements for normal functioning of human body [8,9], being an essential component for successful resistance against viruses including COVID-19 [10]. Recent studies demonstrated that ZnO NPs, together with photocatalytic therapy (irradiation with UVA-1), caused cytotoxicity of human head and neck squamous cell carcinoma in vitro [11]. In cosmetology, ZnO nanomaterials has proved to be effective in UV-protective creams [12,13]. Nanocomposites of polymers reinforced with ZnO NPs to improve their mechanical and antibacterial properties and capable of absorbing UV light can be used in food packaging [14,15]. Polymers incorporated with ZnO also demonstrate better mechanical properties and shape-memory effect, which makes them attractive for 3D printing [16]. When embedded into polymer, ZnO NPs can provide additional protection against corrosion with a prolonged release of inhibitor and self-healing ability of its composite coating [17]. ZnO nanomaterial incorporated in polystyrene not only improves the quality of film, making it a denser, smoother and uniform layer for electron transfer in solar cells, but also increases contact area between hydrophilic ZnO and the hydrophobic active layer [18].

Polymer composites based on ZnO and fibers produced via electrospinning were found to exhibit improved antimicrobial effect when compared with conventional materials [19,20] and demonstrated a wide range of applications as filters [21], bandage materials [22], and tissue frames [23]. Depending on potential applications, there are several methods of preparing composites of polymers with ZnO [22], such as fiber post-treatment after electrospinning [19], coaxial electrospinning to give rise to a core-shell fiber structure, encapsulation of active agent before mixing it with solution for electrospinning, or mixing active agent with its polymer solution immediately before electrospinning procedure. In Ref. [20], the authors compared mechanical properties and antibacterial activity of PLLA/ZnO composites obtained by electrospinning of ZnO dispersion in PLLA solution and by a combination of electrospinning of polymer solutions with electrospraying of nano-ZnO dispersions.

Virovska and coauthors compared photocatalytic activity and antibacterial action of PLLA/ZnO produced via electrospinning with ZnO NPs, which were either embedded into the fibers or localized on their surface [24]. Antibacterial activity was shown to be more pronounced when ZnO nanomaterial was located on the material surface [20,24]. However, in some cases, prolonged release of ZnO is needed, for instance for controlling cell differentiation [25]. It should be added that so far, the maximum concentration of ZnO NPs loaded into PLLA via electrospinning was 23 wt.% [24]. Typically, for testing mechanical and antibacterial properties, PLLA/ZnO composites with low NP loads (below 5 wt.%) were reported [20,25]. Thus, investigation of PLLA/ZnO membranes with various ZnO loads is a timely task which will permit to understand better the structure, properties and behavior of such membranes, as well as possible areas of their application.

In the present work, we prepared and investigated composite membranes of PLLA incorporated with antibacterial ZnO NPs, which are potentially promising for wound bandaging and as adhesive pads in regenerative medicine. The main purpose of this research was to study the effect of the content of incorporated ZnO NPs on physicochemical properties and bactericidal effect of the produced membranes. The novelty and originality of this work are primarily underpinned with the following: (1) NPs of ZnO with «pure» (non-modified and not-passivated) surface were prepared via laser ablation in air, which provided us with a unique ZnO nanomaterial. (2) Composite membranes with ZnO loads as high as up to 40 wt.% were prepared via electrospinning of solution that contained dispersed nanoparticles. This allowed us to study the mechanical and antibacterial properties of biomedical membranes with a wider range of embedded ZnO agent.

## 2. Materials and Methods

### 2.1. Preparation of PLLA/ZnO Composite Membranes

PLLA/ZnO composite membranes were electrospun from polymer solutions containing ZnO NPs prepared as nanopowder by means of the pulsed laser ablation in air technique. Nanosecond pulsed Nd:YAG laser was applied, with wavelength, pulse duration, pulse energy and frequency being 1064 nm, 7 ns, 150 mJ, and 20 Hz, respectively [19]. The use of this method, with Zn as target ablated in air and with no other precursors, permitted to generate ZnO NPs with pure and active surface, which is believed to be promising for biomedical applications [19]. The dispersions were prepared by mixing 0, 0.3, 0.6, 1.2 or 2.4 g of ZnO NPs in 93 g of chloroform in a glass reactor for 10 h at room temperature until a uniform white liquid was achieved. Then, 7, 6.7, 6.4, 5.8, or 4.6 g of poly-L-lactic acid (PL18, Corbion Purac, Netherlands) was added, after which the product was magnetically stirred for another 10 h at room temperature to achieve a uniform viscous white liquid.

40 mL of as-prepared polymer dispersion was electrospun in a typical procedure using a NANON-NF-01 electrospinning setup (MECC Co., Ltd., Fukuoka, Japan), with working parameters being as follows: a flow of polymer solution of 1 mL/min, voltage of 27 kV, cylindrical collector speed of 50 rpm, and a needle-collector distance of 17 cm. The formed material was heat-treated to get rid of residual organic solvent. For this, it was placed in a drying cabinet, where it was heated to a temperature of 100 °C and kept at this temperature for 12 h. The obtained material was cooled to room temperature and placed in sealed plastic bags. Thus, samples with ZnO load of 0, 5, 10, 20, and 40 wt.% were fabricated (hereafter denoted as PLLA, PLLA/ZnO_5%, PLLA/ZnO_10%, PLLA/ZnO_20%, and PLLA/ZnO_40%, respectively).

### 2.2. Characterization of PLLA/ZnO Composite Membranes

The surface morphology of PLLA and PLLA/ZnO composite membranes was studied by scanning electron microscopy (SEM, VEGA 3 SBH, Tescan, Brno, Czech Republic). Energy dispersive X-ray spectroscopy (EDX, AztecLive Lite Xplore 30, Oxford Instruments, UK) was used for elemental analysis. Obtained SEM images were analyzed by using the Image J software (National Institutes of Health, USA).

The crystal structure of PLLA and PLLA/ZnO composite membranes was analyzed by X-ray diffractometry (XRD), for which a XRD 6000 model (from Shimadzu, Kyoto, Japan) was used. Phase identification and quantitative analysis of XRD patterns were conducted using the database PDF4.

The water contact angle was measured via the sessile drop method using a contact angle goniometer (EasyDrop, Krüss, Germany). At least five measurements were carried out on different surface sites, after which their results were averaged. The surface density of the membranes was measured following the standard procedures previously described elsewhere [26]. Three samples sized as 10 × 10 cm^2^ were taken, weighed, while their thickness was measured. The density was evaluated as the mass per volume [26].

Strength and elongation of PLLA and PLLA/ZnO composite membranes with sizes of 60 × 20 mm^2^ and thickness of 166 ± 20 μm were studied using an Instron 3369 material testing machine (Instron, model 2519-102, USA) equipped with a 50 N load cell at a traverse speed of 10 mm/min. At least five samples were tested for each composition.

Fourier-transform infrared (FTIR) spectra were registered by a spectrometer (Nicolet 6700, Thermo Fisher Scientific, Waltham, MA, USA). UV-Vis absorption spectra of powder ZnO, as well as of PLLA and PLLA/ZnO composite membranes, were examined by the diffuse-reflection spectroscopy (DRS) technique on a Cary 100 spectrophotometer (Varian, Australia) with accessory DRA-CA-30I (Labsphere, USA) in the range of 200–800 nm. The obtained absorption spectra were used to evaluate the bandgap values (E_g_) of the ZnO powder and PLLA/ZnO composite membranes in accordance with the Tauc method [27]. Photoluminescence (PL) spectra of the samples were recorded at room temperature by means of spectrofluorometer (CM 2203, Solar, Belarus) with an excitation wavelength of 310 nm.

### 2.3. Antibacterial Activity of PLLA/ZnO Composite Membranes

Antibacterial activity of the prepared composite membranes was tested in accordance with the standard ISO 20743:2013 [28]. The method compares growth of bacteria on the control sample (sample PLLA in this study) and on the tested ones (PLLA/ZnO membranes with different ZnO content in our case). The tests were conducted with two bacterial strains: the gram-positive *S. aureus* (test strain ATCC 25923) and gram-negative *E. coli* (test strain B-6954, Russian Collection of Microorganisms). Both the methodology and conditions of the experiments were previously presented in greater detail elsewhere [19]. The value of antibacterial activity (*A*) was determined as:

*A* = (lg*C*_t_ − lg*C*_0_) − (lg*T*_t_ − lg*T*_0_) = *F* − *G* (1)

where *F* and *G* are the values observed on the control and ZnO-loaded PLLA membranes, respectively; *C*_0_, *C*_t_, *T*_0_ and *T*_t_ are the average number of bacteria (CFU/mL) obtained on three control samples (*C*) or on three ZnO-loaded ones (*T*) immediately after inoculation (*C*_0_ and *T*_0_) and after 24 h of incubation (*C*_t_ and *T*_t_). Note that in this model, for *T*_t_ ≤ 1, the value of lg*T*_t_ is considered equal to 0.

Depending on the experimentally determined value of *A*, the tested material is treated as either bacteriostatic (*C*_t_ > *T*_t_) or bactericidal (*T*_t_ = 0). Taking into account that the bactericidal effect implies the death of all bacteria during the incubation and that *C*_0_ ≈ *T*_0_, formula (1) for the maximum value of experimental *A* can be modified and presented as Formula (2):

*A_max_* ≈ lg*C*_t_ (2)

## 3. Results

### 3.1. Physico-Chemical Properties of PLLA/ZnO Composite Membranes

**Figure 1:**
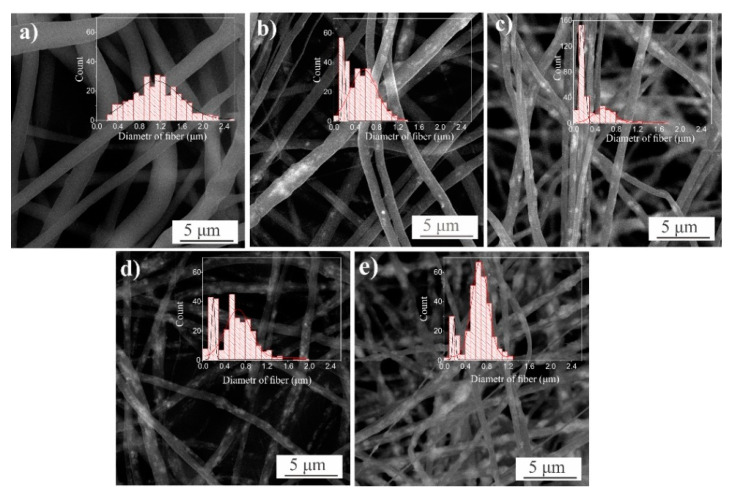
SEM images of PLLA (**a**) and PLLA/ZnO (**b**–**e**) membranes, with fiber diameters presented as histograms. ZnO NPs are loaded into the samples as: 0 (**a**), 5 (**b**), 10 (**c**), 20 (**d**), and 40 (**e**) wt.%.

Figure 1 presents SEM images of samples PLLA (a) and PLLA/ZnO with different ZnO loading (b–e), with corresponding histograms that present fiber size distribution shown as insets. It is seen that the initial PLLA membranes were formed as randomly crossed fibers, the latter fibers having diameters of 1.1 ± 0.24 μm. Incorporation of ZnO NPs into PLLA is seen to result in a decrease of fiber diameters down to 40% of those in the unloaded sample in panel (a). In addition, the distribution of diameters in the ZnO-loaded membranes becomes bimodal, as they tend to have sizes about 0.2 μm, and from 0.5 to 0.7 μm (see Figure 1 and Table 1). It is also well seen in panels (b–e) that the number of ZnO NPs is visually larger along their increase in the initial solution used for electrospinning.

At the same time, the number of visual defects (often observed as aggregates) in the fibers incorporated with ZnO NPs is seen in Figure 1 to grow as the membranes are doped with heavier loads of nanomaterial (see panels (b) to (e)). Because the viscosity of starting polymer with NPs is reduced when more NPs are added, the average diameter of the electrospun fibers they give rise to should become lower. This agrees well with what one can see in Figure 1. It should be noted that there is no linear relationship between the concentration of added ZnO NPs and the decrease in diameters of produced fibers, which is probably explained by the fact that the conductivity of the starting polymer solution changes along its viscosity as more nanomaterial is added as filler [29].

**Figure 2:**
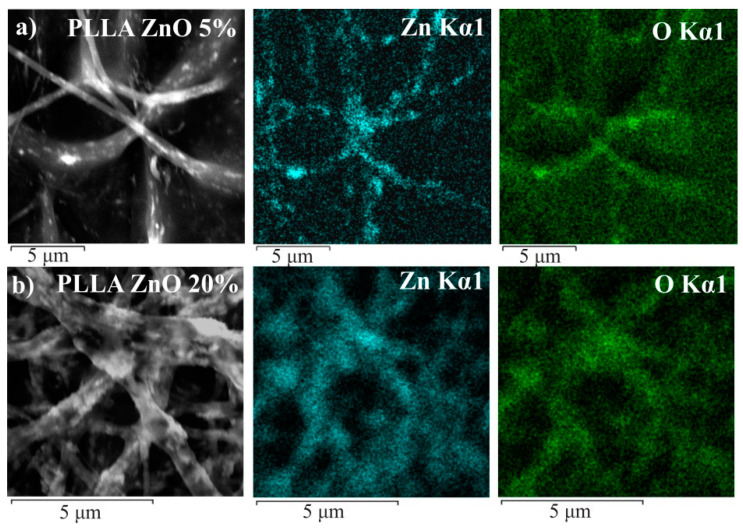
SEM images and corresponding EDX mapping for distribution of zinc (Zn Kα1) and oxygen (O Kα1). Data are presented for samples PLLA/ZnO_5% (**a**) and PLLA/ZnO_20% (**b**).

Figure 2 shows the SEM images and corresponding EDX maps of zinc (ZnKα) and oxygen (OKα) distribution obtained for samples PLLA/ZnO_5% (a) and PLLA/ZnO_20% (b). Note that the distribution of oxygen is somewhat more uniform in the fibers than that of Zn, which is explained by O atoms present in both ZnO NPs and in the polymer. Fairly even distribution of ZnO NPs in PLLA fibers is observed, with ZnO agglomerates observed occasionally. Interestingly, increase in ZnO load from 5 to 20 wt.% was found to result in the enrichment of the fiber surface with zinc oxide, which is seen in both SEM images (left-most images in Figure 2) and in those with Zn and O mapping. Distribution of carbon atoms is not shown in Figure 2 since the samples were analyzed on C-based substrates, making such results less informative. More detailed results obtained for all samples are presented in Supplementary Materials Figure S1.

**Figure 3:**
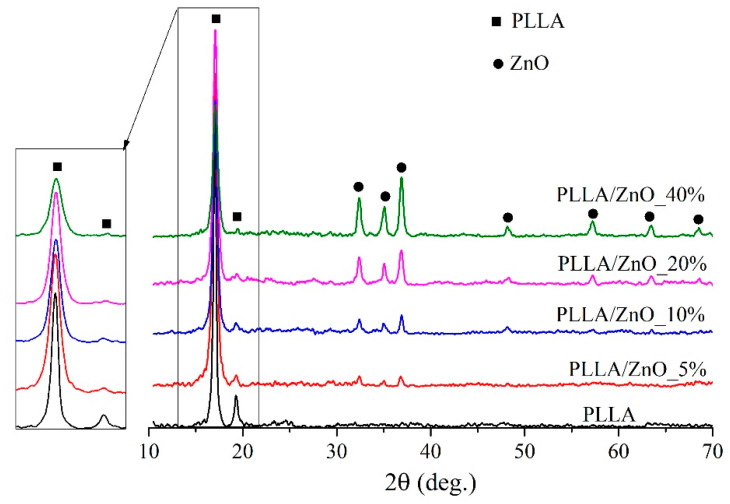
X-ray diffraction patterns of PLLA and PLLA/ZnO composite membranes.

Influence of ZnO NPs on the crystalline state of the electrospun fibers of PLLA was studied by means of XRD, with XRD patterns of undoped and ZnO doped PLLA membranes shown in Figure 3. The pattern of pure PLLA membrane (black line) is seen to exhibit two distinct diffraction maxima and around 17.1° and 19.3°, which correspond to the (200)/(110) and (203) planes of orthorhombic structure, respectively. Upon adding 5 wt.% of ZnO filler, the peaks of wurtzite ZnO are seen gradually to emerge in sample patterns, with their intensity increasing along the amount of incorporated ZnO NPs. At the same time, as well seen in the inset to Figure 3, the peaks of PLLA gradually decrease in intensity (see red, blue, pink, and green patterns that correspond to 5, 10, 20, and 40 wt.% of ZnO NPs). This observation clearly indicates that the increased content of incorporated ZnO NPs hinders crystallization of PLLA as NP aggregates limit the mobility of polymer chains [30].

Physical and mechanical properties of the prepared membranes are compared in Table 1. It is seen that the water contact angle of the pure PLLA membrane was 122° ± 1°, which is explained by the absence of any hydrophilic sites on its surface [31]. Increase in the amount of incorporated ZnO in the range of 5 to 20 wt.% is seen to not cause any considerable changes in the material’s wettability, which is probably explained by the fact that ZnO NPs were embedded into the bulk of PLLA fibers (see Figure 1, panels (b)–(d)). However, further rise of the filler’s load to 40 wt.% is seen in Table 1 to result in enhanced wetting properties (lower water contact angle) of sample PLLA/ZnO_40%. This could be due to the presence of a larger number of ZnO NPs on the fiber surface, which agrees well with the above presented SEM and EDX results for another ZnO-rich sample, PLLA/ZnO_20%. ZnO is well known as a hydrophilic material, and thus the appearance of patches with ZnO is expected to improve the polymer’s hydrophilicity [19]. Previously, a similar decrease in water contact angle for polycaprolactone membranes filled with ZnO NPs was reported in [30].

Both strength limit and elongation at fracture were also determined for all prepared membrane samples. Addition of ZnO NPs in the amount of 10 wt.% is seen in Table 1 to lead to strength values up to 1.4 larger in comparison with that of the non-doped PLLA membrane (Table 1, column 7). There are three possible reasons for this finding. (1) The homogeneous dispersion formed at low ZnO concentrations in PLLA matrix leads to a uniform distribution of stresses in the fibers, as well as to minimal number of stress centers where stresses are transferred from the polymer to the filler [32]. (2) ZnO NPs are known to reinforce composites with polymer through formation of hydrogen bonds between the polymer (PLLA fibers in our case) and hydroxyl groups of ZnO NPs [20]. (3) Increase in the content of ZnO within the range of 5–10 wt.% leads to an increase in density of the formed membranes, along with a decrease in the average fiber’s diameter (Table 1). As a result, the volume of such ZnO-doped membranes is formed by a larger number of fibers with a smaller diameter, which leads to reduced specific load on each fiber. In turn, this leads to a higher total strength of the membrane.

Incorporating more ZnO NPs into the PLLA matrix (in the range of 20–40 wt.%) was found to result in ZnO agglomerates formed in the matrix (see Figure 1d,e). Consequently, along with a further decrease of fibers’ diameter and PLLA crystallite sizes, this led to the formation of defects that acted as stress centers, which inhibit stress transfer between the polymer matrix and the filler (ZnO NPs). As seen in Table 1 (column 7), this resulted in a reduction of total membrane strength.

**Figure 4:**
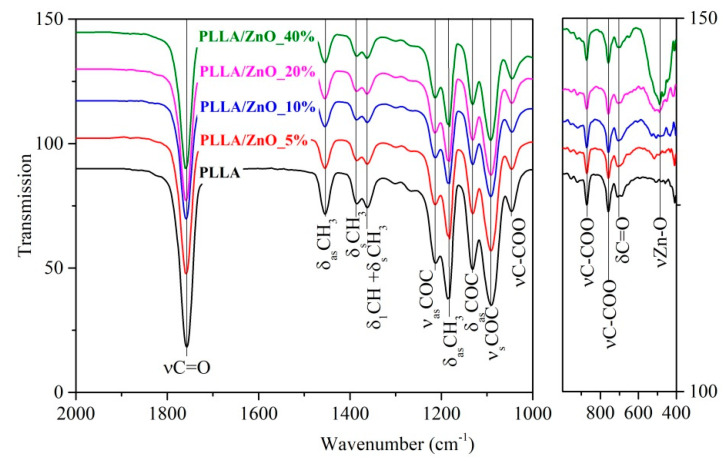
FTIR spectra of PLLA and ZnO-loaded PLLA membranes.

Figure 4 presents FTIR spectra of PLLA and PLLA/ZnO membranes, with the main bands belonging to polymer PLLA [33,34]. The strongest peak located at around 1754 cm^−1^ corresponds to the vibration of the C = O bond, while symmetric and asymmetric bending vibration of CH_3_ groups were observed at 1454, 1385, and 1362 cm^−1^. The bands seen at 1213–1132 cm^−1^ are assigned to valent and deformational asymmetric vibrations of bonds in the COC and CH_3_ groups. The peak with maximum at 1092 cm^−1^ is related to valent symmetric vibrations of the COC bonds, whereas valent vibrations of bonds C-CH_3_ and C-COO manifest themselves at 1046 and 870 cm^−1^, respectively. Finally, the peak in the region from 550 to 430 cm^−1^ appears for samples incorporated with ZnO NPs and corresponds to the Zn–O bending vibrations [35].

It is seen in Figure 4 that the increased amount of ZnO NPs doped into PLLA leads to higher intensity of the peak observed between 550 and 430 cm^−1^, which confirms that more nanomaterial was embedded as filler. Note that at elevated loads of ZnO NPs, the FTIR spectra of PLLA membranes do not exhibit any new lines or line shifts. This implies that the polymer-ZnO interaction is purely based on the Van der Waals forces with no covalent bonds forming. As the intensity of peak at around 1362 cm^−1^ decreases, this confirms that the incorporation of ZnO nanomaterial hinders crystallization in PLLA matrix [36].

**Figure 5:**
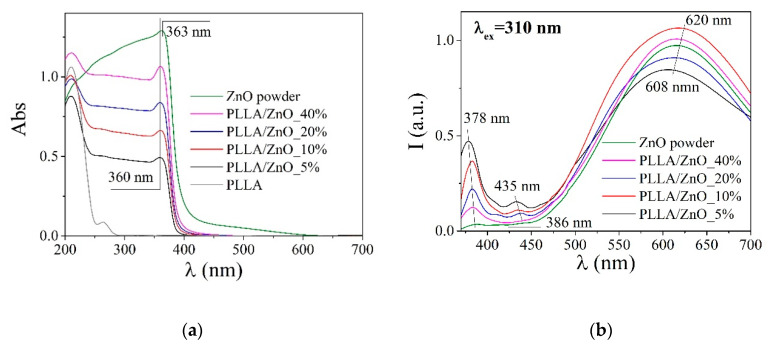
Absorption (**a**) and PL (**b**) spectra of prepared composite membranes. Spectra of laser-prepared ZnO NPs are given for comparison.

Spectral and luminescent properties of all prepared samples are presented in Figure 5 (where similar spectra are also given for the laser-prepared ZnO NPs) and in Table 2. The absorption spectrum of ZnO NPs is seen in Figure 5a (green line) to have a strong maximum at 363 nm, which is associated with the electron transition from the valence band to the conduction band. The bandgap of the used ZnO nanomaterial was thus evaluated to be E_g_ = 3.22 eV, being slightly lower than that of bulk ZnO (3.3 eV) [37]. The observed small difference can be explained by either the size effect or oxygen vacancies that introduce new states in the band gap of ZnO NPs laser-generated in air [38].

Upon incorporating ZnO NPs into PLLA polymer, the polymer samples are seen in Figure 5a to demonstrate a small blueshift (by 3 nm) of their absorption edge, which corresponds to larger values of their bandgap compared with the ZnO nanomaterial (Table 2). This finding is explained by coating of the semiconducting material with polymer [39].

The PL spectrum of the ZnO powder used as an antibacterial agent is seen in Figure 5b to show a small exciton peak at 378 nm and a wide band at around 620 nm, the latter band being related to various defects in crystal structure of ZnO [19,40,41]. Meanwhile, in the ZnO-incorporated PLLA membranes, the peak at 380 nm was found to redshift, while the relative intensity of their defect-related emission decreased. This finding can be explained by surface passivation of ZnO nanomaterial embedded into the polymer. This effect is the most pronounced for sample PLLA/ZnO_5% where the exciton-related peak at 378 nm is best seen in Figure 5b (black line) [42].

### 3.2. Antibacterial Properties of PLLA/ZnO Composite Membranes

**Figure 6:**
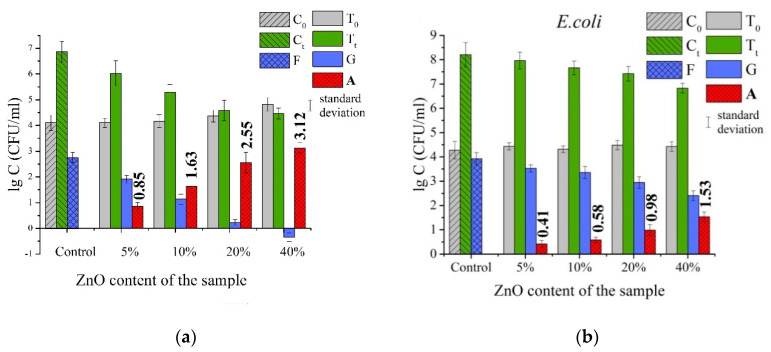
Results of antibacterial tests according to the ISO 20743:2013 standard obtained for *S. aureus* (**a**) and *E. coli* (**b**) bacteria.

Figure 6 presents the results of antibacterial tests carried out with both non-doped PLLA and samples incorporated with ZnO NPs and with two bacterial strains: gram-positive *S. aureus* (Figure 6a) and gram-negative *E. coli* (Figure 6b). The methodology used was based on the standard ISO 20743:2013, implying a direct contact of sample with a bacterial medium. Figure 6 compares the logarithmic values of the number of bacteria grown on the control sample (*C*, pure PLLA membrane in our case) and samples with antibacterial agent (*T*, four PLLA/ZnO samples in our case) for the initial contact (*C*_0_ and *T*_0_, contact time 0 h) and after prolonged contact (*C*_t_ and *T*_t_, contact time of 24 h). According to the methodology [28], the antibacterial effect is observed when bacterial growth on a tested sample with antibacterial agent (*G*, blue bars) is lower than that on the control sample (*F*, blue bars with shading). Comparison of the green bars in Figure 6, corresponding to control sample (*C*_t_, green bars with shading) and those with ZnO NPs (*T*_t_, green bars), clearly demonstrates that bacterial growth was suppressed on all ZnO-incorporated membranes, which was observed for both the *S. aureus* and *E. coli* strains (Figure 6).

Hence, based on Figure 6 one can conclude that for all ZnO-loaded membranes, antibacterial action was observed, which is manifested by inhibition of bacterial growth on such samples (for both tested bacteria strains). Note that an antibacterial agent is considered as bactericidal (when it kills bacteria), or as bacteriostatic (when it inhibits their growth). In the former case, the *G* parameter in formula (1) must be *G* < 0 (i.e., *T*_t_ < *T*_0_), while in the latter case, it should be within the following range: *F* > *G* ≥ 0. Obvious bactericidal behavior was only found for sample PLLA/ZnO_40% towards *S. aureus*, i.e., the concentration of bacteria on its surface after incubation period was lower than that on the control sample, ZnO-free PLLA (*T*_t_ < *T*_0_). It is seen in Figure 6a,b that the value of antibacterial activity (*A*, red bars in panels (a,b)) rises along with the amount of ZnO NPs loaded into PLLA fibers. It is noteworthy that antibacterial activity (seen as red bars in panels (a,b)) is considerably higher towards *S. aureus* when compared with the results observed for *E. coli*. This is believed to be related with the difference in the structure of membranes in these two bacteria [19,43]. The cell membrane of the *E. coli* is known to have a more complex structure, being thicker and chemically different from that of the *S. aureus* [19,43].

There are several mechanisms explaining antibacterial action of ZnO NPs [44]: through the formation of reactive oxygen species (ROS), release of Zn^2+^ ions via partial ZnO dissolution, electrostatic sticking of ZnO NPs to the bacterium membrane, and so on. All these mechanisms result in the decay of the bacterium cell membrane, thus causing its disfunction and penetration of destructive antibacterial species, such as ROS and Zn^2+^, inside the cell. Note that the process of internalization (i.e., absorption of the NP by the bacterium cell) is governed by the size, chemistry, morphology, defects, and even functionalization of the ZnO NP. For example, antibacterial activity of ZnO NPs was found to rise for smaller NPs since such particles have a larger surface area [45,46]. Besides, smaller ZnO NPs are known to have larger reactivities because of a greater number of forming ROS whose generation is also dependent on particle’s surface area [47,48].

**Figure 7:**
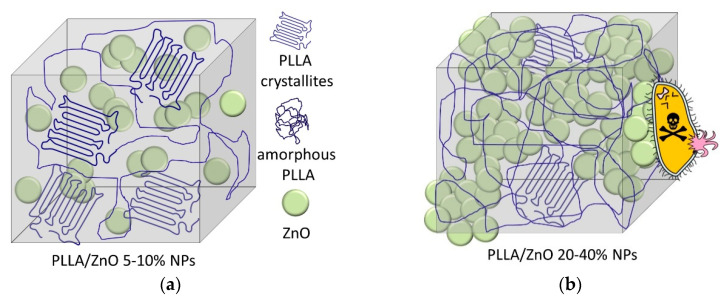
Schematic presentation of PLLA/ZnO membranes doped with smaller (**a**) and larger (**b**) amounts of ZnO NPs. ZnO NPs are distributed uniformly in the bulk of material presented in panel (**a**), while they were agglomerated and partially protrude on the surface of PLLA matrix in panel (**b**).

Our previous studies showed that similar laser-generated ZnO NPs, when deposited on the surface of PLLA matrix, demonstrated high bactericidal action towards *S. aureus* and high bacteriostatic action towards *E. coli* bacteria [19]. In the present work, ZnO NPs were embedded into PLLA fibers, which limited access to the active surface of the NPs (Figure 7). This was expected to guarantee that such NPs would remain inside PLLA fibers without penetrating the treated tissue (e.g., inside the wound), providing a slower but longer release of Zn^2+^ ions and/or generation of ROS. At the same time, we also aimed at providing a biomedical material with primarily bacteriostatic effect (i.e., minimized bactericidal action), which inhibits bacterial growth and lets the host body act properly and kill bacteria through its own mechanisms.

Thus, the obtained results showed that the electrospun PLLA membranes with 5–10 wt.% of ZnO NPs possessed better mechanical properties in comparison with their counterpart based on pure PLLA. This is believed to be due to uniform distribution of ZnO NPs and PLLA crystallites inside the composite fibers (Figure 7a). In this case, only small patches on fiber surface were found where ZnO material is exposed, which is why such samples demonstrated lower bacteriostatic behavior. In contrast, at higher loading amounts of ZnO NPs (20–40 wt.%), the NPs tend to agglomerate, which prevents polymer’s crystallization and leads to poorer mechanical properties of such membranes. At the same time, such ZnO-rich PLLA fibers have more ZnO NPs protruding from the fiber bulk (Figure 7b), which is why such membranes demonstrated a higher antibacterial effect.

## 4. Conclusions

Polymer-based composites incorporated with inorganic nanoparticles produced via electrospinning are promising materials for nanomedicine. The present study used this inexpensive and efficient method to prepare composite membranes of poly-L-lactic acid (PLLA, or polylactide) incorporated with ZnO nanoparticles (NPs) generated via pulsed laser ablation of metallic Zn in air. Importantly, for the first time, this work prepared and tested PLLA membranes with as high content of incorporated ZnO NPs as 5–40 wt.%. The following trends were observed when the amount of incorporated ZnO NPs was increased:

(i) Mechanical properties of the composite materials were enhanced for ZnO contents as high as 10 wt.%, after which the material’s strength was found to deteriorate. This is believed to be related to the NP-polymer interactions, as well as to particle distribution inside electrospun PLLA fibers. At high loads of ZnO (20–40 wt.%), the incorporated NPs tended to agglomerate and crystallization of PLLA crystallites inside the fibers was hindered.

(ii) The antibacterial activity of the composite membranes increased monotonously. The as-prepared composites demonstrated bacteriostatic action towards the *S. aureus*, while this effect was somewhat lower towards the *E. coli* bacteria. At the maximum studied content of incorporated ZnO NPs (of 40 wt.%), the PLLA/ZnO membrane exhibited a bactericidal effect towards the *S. aureus* bacteria.

The prepared composite membranes are believed to find applications as antibacterial dressings with prolonged action for treatment of purulent-inflammatory diseases of the skin and other soft tissues.

## Figures and Tables

**Table 1:** Physical and mechanical characteristics of the as-prepared membranes (average ± standard deviation).

Sample	Density, g/cm^3^	Average Fiber Diameter, μm (Peak Intensity)		Water Contact Angle H_2_O, °	Stretch, %	Strength Limit, MPa
PLLA	0.15	1.1 (30)		122 ± 1	62 ± 5	2.7 ± 0.1
PLLA/ZnO_5%	0.16	0.2 (40)	0.6 (40)	124 ± 2	35 ± 1	3.7 ± 0.2
PLLA/ZnO_10%	0.17	0.2 (150)	0.6 (30)	126 ± 2	30 ± 4	3.9 ± 0.2
PLLA/ZnO_20%	0.16	0.2 (30)	0.7 (70)	127 ± 1	44 ± 2	3.1 ± 0.2
PLLA/ZnO_40%	0.19	0.2 (60)	0.5 (35)	117 ± 1	48 ± 7	2.5 ± 0.2

**Table 2:** Spectral and luminescent properties of the obtained materials.

Sample	Luminescence		ΔE, eV
	λ_max_, nm	I_inter-band_/I_defect_^1^	
PLLA	431	-	-
PLLA/ZnO_5%	379/435/612	0.5	3.27
PLLA/ZnO_10%	382/431/612	0.3	3.27
PLLA/ZnO_20%	382/439/608	0.2	3.27
PLLA/ZnO_40%	382/607	0.08	3.27
ZnO powder	387/608	0.04	3.22

^1^ The ratio of the intensity of interband and defect luminescence.

## Supplementary Materials

The following are available online at https://www.mdpi.com/1996-1944/14/1/2/s1, Figure S1: SEM images and corresponding EDX maps of carbon (C Kα1), zinc (Zn Kα1), and oxygen (O Kα1) obtained for the samples.
