# Supplementary material for: Structure and Properties of Biodegradable PLLA/ZnO Composite Membrane Produced via Electrospinning
Source: Materials (Basel). 2020 Dec 22;14(1):2. doi: 10.3390/ma14010002 (PMC7792573; doi:10.3390/ma14010002)

materials

Article

Structure and Properties of Biodegradable PLLA/ZnO Composite Membrane Produced via Electrospinning

Daria A. Goncharova [1,*], Evgeny N. Bolbasov [2], Anna L. Nemoykina [3], Ali A. Aljulaih [4,5], Tamara S. Tverdokhlebova [2], Sergei A. Kulinich [4,6,7,*] and Valery A. Svetlichnyi [1]

1 Laboratory of Advanced Materials and Technology, Tomsk State University, Tomsk 634050, Russia; v_svetlichnyi@bk.ru
2 Laboratory of Hybrid Plasma Systems, National Research Tomsk Polytechnic University, Tomsk 634050, Russia; ebolbasov@gmail.com (E.N.B.); aramat_tts@mail.ru (T.S.T.)
3 Laboratory of Biopolymers and Biotechnology, Tomsk State University, Tomsk 634050, Russia; nemoykina@rambler.ru
4 Department of Mechanical Engineering, Tokai University, Hiratsuka, Kanagawa 259-1259, Japan
5 Division of Physical Science and Engineering, King Abdullah University of Science and Technology (KAUST), Thuwal 23955-6900, Saudi Arabia; ali.julaih@kaust.edu.sa
6 Research Institute of Science and Technology, Tokai University, Hiratsuka, Kanagawa 259-1259, Japan
7 School of Natural Sciences, Far Eastern Federal University, Vladivostok 690091, Russia
* Correspondence: dg_va@list.ru (D.A.G.); skulinich@tokai-u.jp (S.A.K.)

materials

Figure S1. SEM images and corresponding EDX maps of carbon (C Kα1), zinc (Zn Kα1) and oxygen (O Kα1) obtained for the samples.

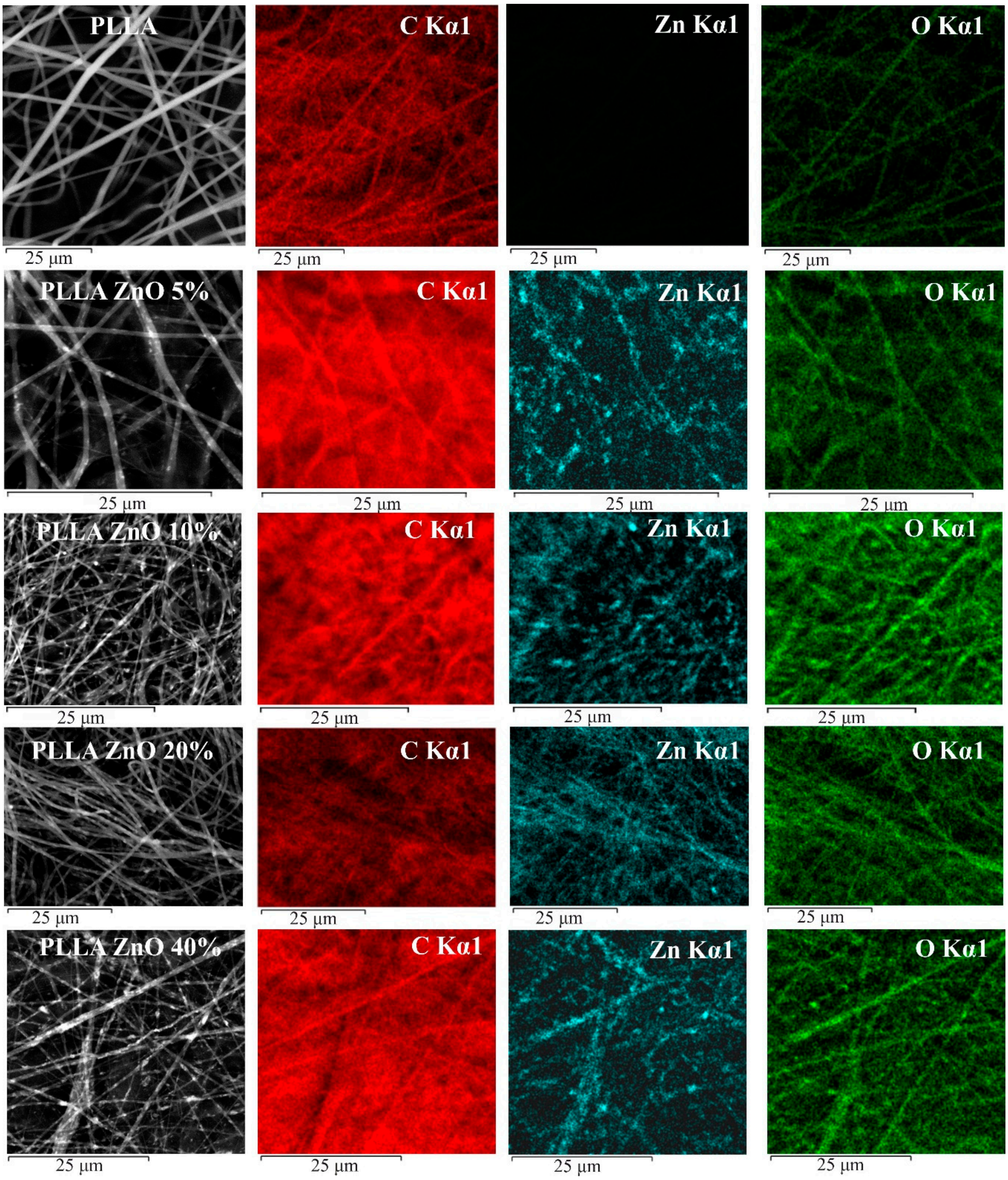

Supplement: Supplementary file 1 [file materials-14-00002-s001.pdf]
